# Histological Evaluation of Insulin-like Growth Factor-I in Experimental Intestinal Ischemia–Reperfusion Injury: A Rat Study

**DOI:** 10.3390/diseases14080300

**Published:** 2026-08-18

**Authors:** Apostolos Andronikou, Dimitra Psalla, Rafail Ioannidis, Apostolos Papalois, Stavros Iliadis, Konstantina Dinaki, Apostolos Kamparoudis

**Affiliations:** 1 5th Department of Surgery, Ippokratio General Hospital, Aristotle University of Thessaloniki, 54642 Thessaloniki, Greece; 2 Department of Surgery, General Hospital of Mytilene, 81100 Mytilene, Greece; 3 Laboratory of Pathology, School of Veterinary Medicine, Aristotle University of Thessaloniki, 54124 Thessaloniki, Greece; 4 Department of Anesthesiology and Pain Medicine, General Hospital of Drama, 66100 Drama, Greece; raphaioan@gmail.com; 5 2nd Department of Surgery, Aretaieion University Hospital, School of Medicine, National and Kapodistrian University of Athens, 11528 Athens, Greece; 6 Laboratory of Biological Chemistry, Department of Medicine, School of Health Sciences, Aristotle University of Thessaloniki, 54124 Thessaloniki, Greece; 7 School of Medicine, Aristotle University of Thessaloniki, 54124 Thessaloniki, Greece

**Keywords:** IGF-I, intestinal ischemia–reperfusion, recombinant insulin-like growth factor-I, rat model, Chiu score

## Abstract

Purpose: Intestinal ischemia–reperfusion injury (IRI) remains a major clinical challenge associated with substantial morbidity. Although the biological functions of insulin-like growth factor-I (IGF-I) have been extensively investigated, evidence regarding the direct administration of exogenous recombinant IGF-I in experimental intestinal IRI remains limited. This study evaluated the histological effects of recombinant IGF-I administered at different time points in a rat model of intestinal IRI. Methods: Male Wistar rats underwent 45 min of superior mesenteric artery occlusion followed by 3 h of reperfusion. Animals were allocated to five groups: Sham, untreated Control, and three IGF-I-treated groups receiving intraperitoneal recombinant IGF-I before ischemia, during ischemia, or at reperfusion. Histological injury was assessed using the Chiu grading system (0–5). Data were analyzed using non-parametric statistical methods. Results: Forty-five rats were included in the final analysis. Untreated controls demonstrated the greatest mucosal injury (median Chiu score: 4, IQR: 3–4), whereas Sham-operated animals exhibited minimal injury (median: 0, IQR: 0–1). All IGF-I-treated groups demonstrated lower median histological injury scores (median: 2), with the lowest median observed in the reperfusion group (IQR: 1–3). However, none of the individual comparisons with untreated controls remained statistically significant after Bonferroni correction. A post hoc exploratory pooled analysis demonstrated a similar histological pattern but likewise did not show a statistically significant difference between pooled IGF-I-treated animals and controls after adjustment for multiple comparisons. Conclusions: In this experimental rat model, exogenous recombinant IGF-I administration was consistently associated with lower histological injury scores than untreated ischemia–reperfusion controls, although a definitive treatment effect could not be established. These findings provide additional experimental evidence regarding the histological effects of recombinant IGF-I in intestinal ischemia–reperfusion injury and support further investigation in larger experimental studies incorporating quantitative histological, molecular, and functional endpoints.

## 1. Introduction

Acute intestinal ischemia–reperfusion represents a major challenge in clinical practice, both diagnostically and therapeutically. Despite decades of research, the pharmacological management of intestinal ischemia–reperfusion remains an area of ongoing investigation. Intestinal ischemia–reperfusion injury (IRI) contributes substantially to postoperative morbidity following intestinal transplantation, mesenteric revascularization, strangulated bowel obstruction, and major vascular surgery. In recent years, substantial research efforts have been directed toward understanding the pathophysiology of intestinal IRI and exploring potential therapeutic interventions. Various approaches, including antioxidant agents, free radical scavengers, glycine-based interventions, adjunctive therapies, and leukocyte-modulating treatments, have been investigated; however, satisfactory results have not yet been achieved. Therefore, the identification of interventions capable of favorably modifying reperfusion-induced intestinal injury remains an important unmet clinical need [1,2,3].

In intestinal tissue, ischemia is associated with interstitial edema, formation of cell-surface blebs, villous epithelial sloughing, and mucosal hemorrhage. If adequate perfusion is not promptly restored, cell death ensues and tissue necrosis may progress transmurally from the mucosa toward the serosa [4,5]. Nevertheless, restoration of blood flow may itself induce additional tissue injury. The structural and functional abnormalities that develop after reperfusion of viable ischemic tissue, and which may exceed those caused by ischemia alone, constitute ischemia–reperfusion injury [3,6].

Insulin-like growth factor-I (IGF-I) is a 7.5 kDa peptide with structural homology to proinsulin that acts as a principal mediator of growth hormone (GH) activity during postnatal life [7]. Its actions are mediated primarily through the type 1 IGF receptor (IGF-1R), a membrane-bound tyrosine kinase receptor. Both IGF-I and IGF-1R are highly conserved across mammalian species; 66 of the 70 amino acids of mature IGF-I are identical in humans, rats, mice, cattle, and pigs [8]. IGF-I stimulates the proliferation of several cell types, including fibroblasts, chondrocytes, osteoblasts, neural cells, smooth and skeletal muscle cells, epithelial cells, and oocytes. In addition to its proliferative actions, IGF-I has been associated with inhibition of cell death during normal development and under conditions of cellular stress or disease [9,10,11].

Although the biological functions of IGF-I have been extensively investigated in experimental models of tissue injury and regeneration, direct evidence regarding the therapeutic administration of recombinant IGF-I in intestinal ischemia–reperfusion remains remarkably limited [12,13]. Most published studies have focused on endogenous activation of the IGF-I signaling pathway or modulation of downstream molecular mechanisms rather than direct administration of recombinant IGF-I [13,14]. Consequently, the direct histological effects of exogenous recombinant IGF-I on intestinal ischemia–reperfusion injury remain insufficiently characterized [12].

GH also exerts important effects on tissue growth and repair; however, many of its biological actions are mediated through IGF-I. Moreover, IGF-I exerts GH-independent effects and directly activates IGF1R-mediated signaling pathways involved in cell survival and proliferation and inhibition of apoptosis [15]. On this basis, IGF-I was selected in the present study to specifically investigate the effects of its direct exogenous administration in intestinal ischemia–reperfusion injury. Given the limited experimental evidence regarding direct recombinant IGF-I administration in intestinal ischemia–reperfusion, the present study aimed to evaluate the histological effects of exogenous recombinant IGF-I administered at different time points relative to ischemia and reperfusion in a standardized rat model.

## 2. Materials and Methods

This study was designed to compare histological intestinal injury among untreated ischemia–reperfusion controls, Sham-operated animals, and three IGF-I administration schedules representing different phases of the ischemia–reperfusion process.

A randomized prospective experimental study was conducted on male Wistar rats in intervention and Control groups. The rats were 12 to 16 weeks old with an average body weight of approximately 300 (250–340) grams. All animals were housed in plexiglass cages under controlled environmental conditions: a temperature between 20 °C and 22 °C, a relative humidity between 55% and 65%, and a 12-h light/12-h dark cycle. The animals had ad libitum access to water and standard rat chow. All animals were of the same strain and age range to minimize biological variability, while for microbiological standardization, conventional laboratory animals were used.

All experiments adhered to national (Greek) and European guidelines for the use of experimental animals and were approved by the local Ethics Committee and the National General Directorate of Agricultural Economy, Veterinary Services & Fisheries, Directorate of Agricultural & Veterinary Policy. The experiments were performed in two series, a pilot study and main study, using a total of 49 Wistar rats (pilot study *n* = 15; main study *n* = 34).

The animals were randomly assigned to five experimental groups in order to investigate the effects of IGF-I administration at different time points relative to the IRI.

### 2.1. Sham Operation Group (Group S)

This group was used to evaluate the effect of anesthesia, laparotomy and preparation of the superior mesenteric artery (SMA). Rats underwent anesthesia and midline laparotomy. The small and large intestines were gently exteriorized and placed on sterile gauze moistened with physiological saline, and the abdomen was left open for 5 min (corresponding to the needed time for SMA preparation and clamping). The intestines were then repositioned into the peritoneal cavity and the abdominal wall was sutured. Forty-five minutes later, the incision was reopened, and 2 min thereafter (corresponding to the clamp removal time) the abdominal wall was resutured.

Three hours later, under anesthesia, a third laparotomy was performed and a 3 cm segment of small intestine located proximally to the ileocecal junction was excised for histological examination. Following the procedure, euthanasia was performed by administration of pentobarbital at a dose of 200 mg/kg (200 mg/mL Sol. Dolethal, Vetoquinol, Lure, France).

### 2.2. Control Group (Group C)

**Figure 1:**
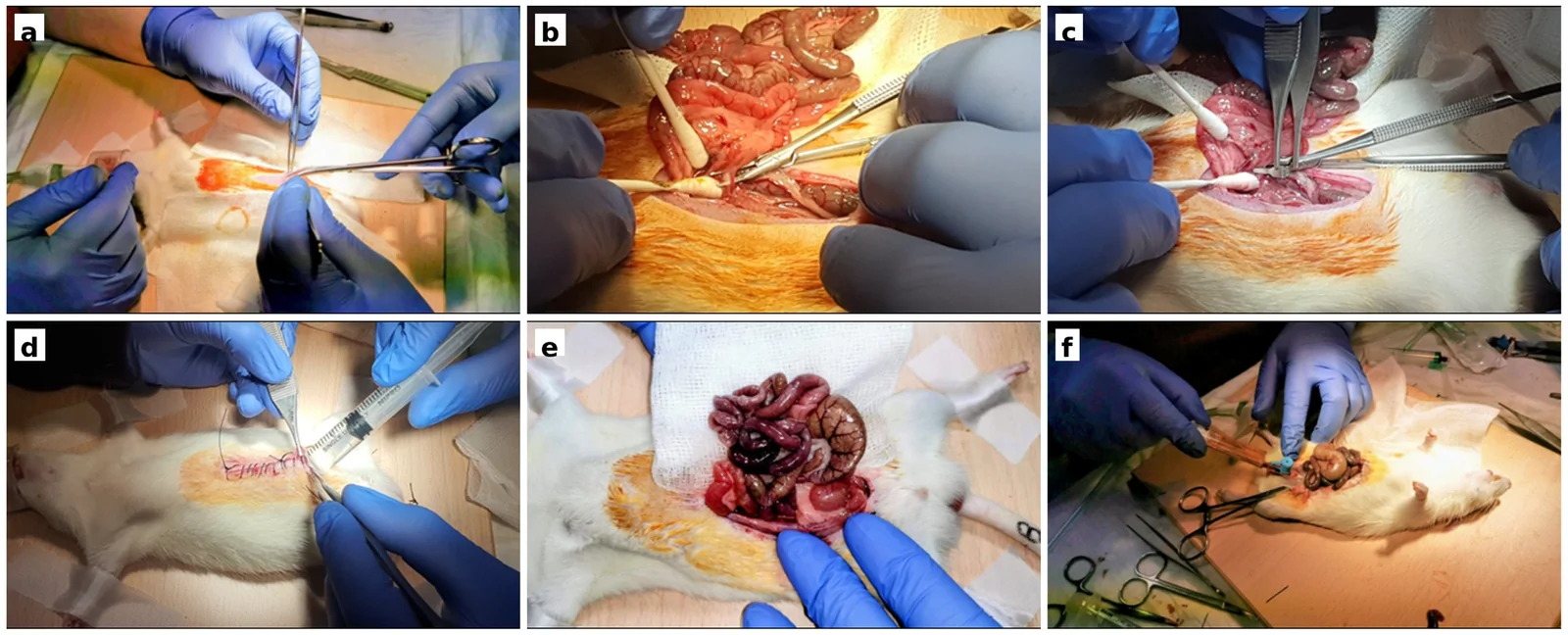
Representative images illustrating the main steps of the experimental surgical procedure. (**a**) First laparotomy, midline incision. (**b**) Dissection of SMA. (**c**) Clamping of SMA. (**d**) Intraperitoneal administration of IGF-I during reperfusion, Group III. (**e**) Third laparotomy, 3 h after reperfusion, preparing bowel for excision. (**f**) Euthanasia by pentobarbital administration.

Rats in this group underwent anesthesia and laparotomy, and the SMA was occluded with a microvascular clamp for 45 min to induce ischemia (Figure 1a–c). A midline laparotomy was performed and the peritoneum was incised. The small intestine was exteriorized and placed on gauze moistened with 0.9% NaCl. The superior SMA was clamped with a microvascular clamp to induce intestinal ischemia. The intestines were repositioned and the abdominal wall was closed with continuous sutures. After the ischemic phase (45 min), the incision was reopened, the clamp was removed to allow reperfusion, and the abdominal wall was resutured.

Three hours later, a third laparotomy was performed for excision of a 3 cm segment proximal to the ileocecal junction for histological analysis (Figure 1e). Euthanasia was then performed by administration of pentobarbital (200 mg/kg) (Figure 1f).

### 2.3. IGF-I Administration Group, 2 h Before Ischemia (Group I)

IGF-I was administered intraperitoneally 2 h prior to the onset of the ischemic phase, followed by the same procedure described for Group C.

### 2.4. IGF-I Administration Group, During Ischemia (Group II)

The same procedure as in Group C was performed, but IGF-I was administered intraperitoneally 30 min after SMA occlusion, during the ischemic phase.

### 2.5. IGF-I Administration Group, During Reperfusion (Group III)

The same procedure as in Group C was performed, but IGF-I was administered intraperitoneally immediately after clamp removal, at the onset of the reperfusion phase (Figure 1d).

### 2.6. Induction to Anesthesia

Prior to surgery, all animals received subcutaneous administration of 1 mL/100 g of 0.9% NaCl, ophthalmic ointment, and preoperative analgesia with ketoprofen at a dose of 5 mg/kg subcutaneously.

### 2.7. Anesthesia

General injectable anesthesia for acute procedures consisted of ketamine (100 mg/kg) and xylazine (20 mg/kg) administered intramuscularly. Supplemental doses equal to one-third of the initial dose were administered every 30–60 min as required to maintain adequate anesthesia.

### 2.8. Surgical Preparation

The abdominal surface was shaved and disinfected, and each rat was positioned and immobilized on the surgical table under sterile conditions.

### 2.9. Surgical Procedures

As described for groups S and C.

### 2.10. IGF-I Administration Protocol

In groups receiving IGF-I (1 mg/mL IGF-I ampule, Prospec, Rehovot, Israel), the compound was administered intraperitoneally as a single dose of 200 μg per 100 g body weight.

### 2.11. Histological Analysis

**Figure 2:**
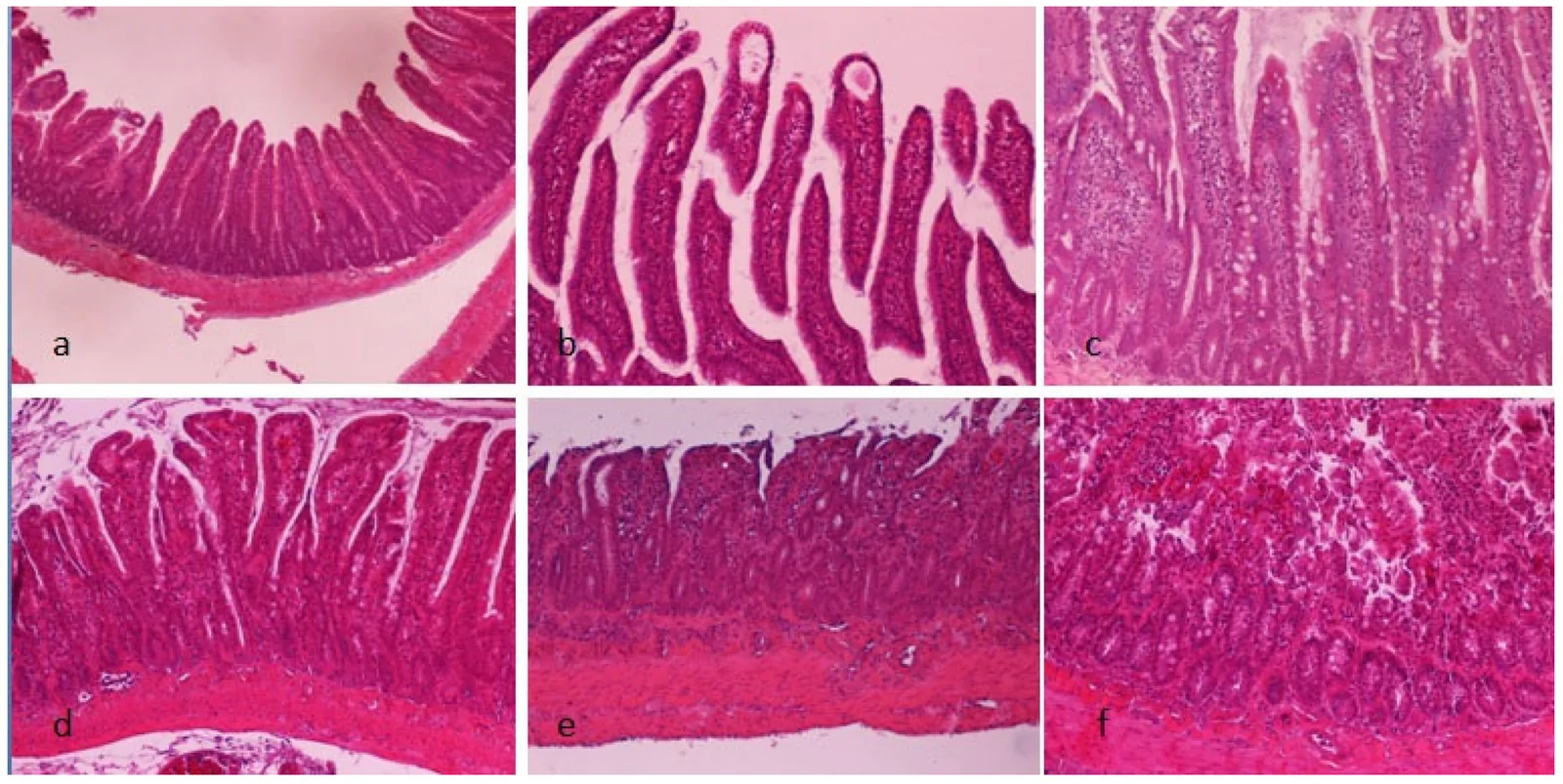
Histological images of the intestines of experimental animals. Hematoxylin–eosin staining. Representative micrographs corresponding to the six different stages of mucosal injury, according to Chiu et al., 1970 [16]. (**a**) Grade 0: normal mucosal villi, magnification ×100. (**b**) Grade 1: development of subepithelial Gruenhagen’s space at the tip of villus, magnification ×200. (**c**) Grade 2: extension of the lamina propria due to edema and infiltration by inflammatory cells, magnification ×200. (**d**) Grade 3: epithelial lifting along the sides of villi and subepithelial accumulation of proteinaceous fluid, magnification ×200. (**e**) Grade 4: denuded villi and expansion of lamina propria due to infiltration by inflammatory cells, magnification ×200. (**f**) Grade 5: epithelial ulceration and disintegration of the lamina propria, magnification ×200. The lower magnification in panel (**a**) was used to provide a broader overview of the preserved mucosal and villous architecture characteristic of Chiu Grade 0.

Tissue samples from the small intestine were fixed in 10% neutral buffered formalin and embedded in paraffin for histological examination. Sections were cut and stained with hematoxylin and eosin (H&E). Histological evaluation was performed by an experienced veterinary pathologist who was blinded to the experimental group allocation. The degree of intestinal mucosal injury was assessed according to the Chiu histological grading system (Grades 0–5), based on the severity of progressive mucosal alterations [16] (Table 1 and Figure 2).

### 2.12. Statistical Analysis

Descriptive data are expressed as medians and interquartile ranges (IQRs) because the Chiu et al. (1970) [16] histological injury score is an ordinal measure (0–5). Normality of distribution was tested using the Shapiro–Wilk test. All treatment groups followed a normal distribution (*p* > 0.05), except for the Sham group (*p* < 0.001). Given the ordinal nature of the data, non-parametric tests were chosen. Comparisons among groups were conducted with the Kruskal–Wallis H test. When the overall test was significant, pairwise Mann–Whitney U tests were performed for post hoc analysis. To adjust for multiple comparisons, a Bonferroni correction was applied, setting the threshold for statistical significance at *α* = 0.005 (0.05/10 pairwise tests). All tests were two-tailed, and a *p*-value < 0.05 was considered statistically significant, except for in the multiple comparisons. All analyses were performed using IBM SPSS Statistics, version 27.0 (IBM Corp., Armonk, NY, USA).

## 3. Results

A pilot study involving 15 rats (3 per group) was conducted to assess the feasibility of the experimental hypothesis and to obtain data for power analysis in order to determine the required sample size for the main study. Two rats were excluded from the statistical analysis: one from Group S died two hours earlier due to possible anesthesia-related complications, and one from Group III due to traumatic bleeding following SMA injury.

In one rat from Group C, failure to clamp the SMA (secondary branch occlusion) and consequent failure to achieve ischemia were identified; however, the experimental procedure was completed and data were collected. In one rat from Group S, pre-existing bowel inflammation was observed during histological analysis.

Following completion of the pilot study, the results and their analysis were submitted to the Protocol Evaluation Committee. The main study was subsequently approved with an additional 34 animals, distributed as follows: Group I (*n* = 6), Group II (*n* = 6), Group III (*n* = 7), Group C (*n* = 7), and Group S (*n* = 8). During the main experimental series, two animals did not complete the procedure and were excluded from the protocol: one animal from Group I died immediately after anesthesia and could not be resuscitated, and one animal from Group III died due to progressive respiratory distress shortly after the second laparotomy and reperfusion.

A total of 45 rats were included in the final histological analysis (Group I, *n* = 8; Group II, *n* = 9; Group III, *n* = 8; Control, *n* = 10; Sham, *n* = 10).

**Figure 3:**
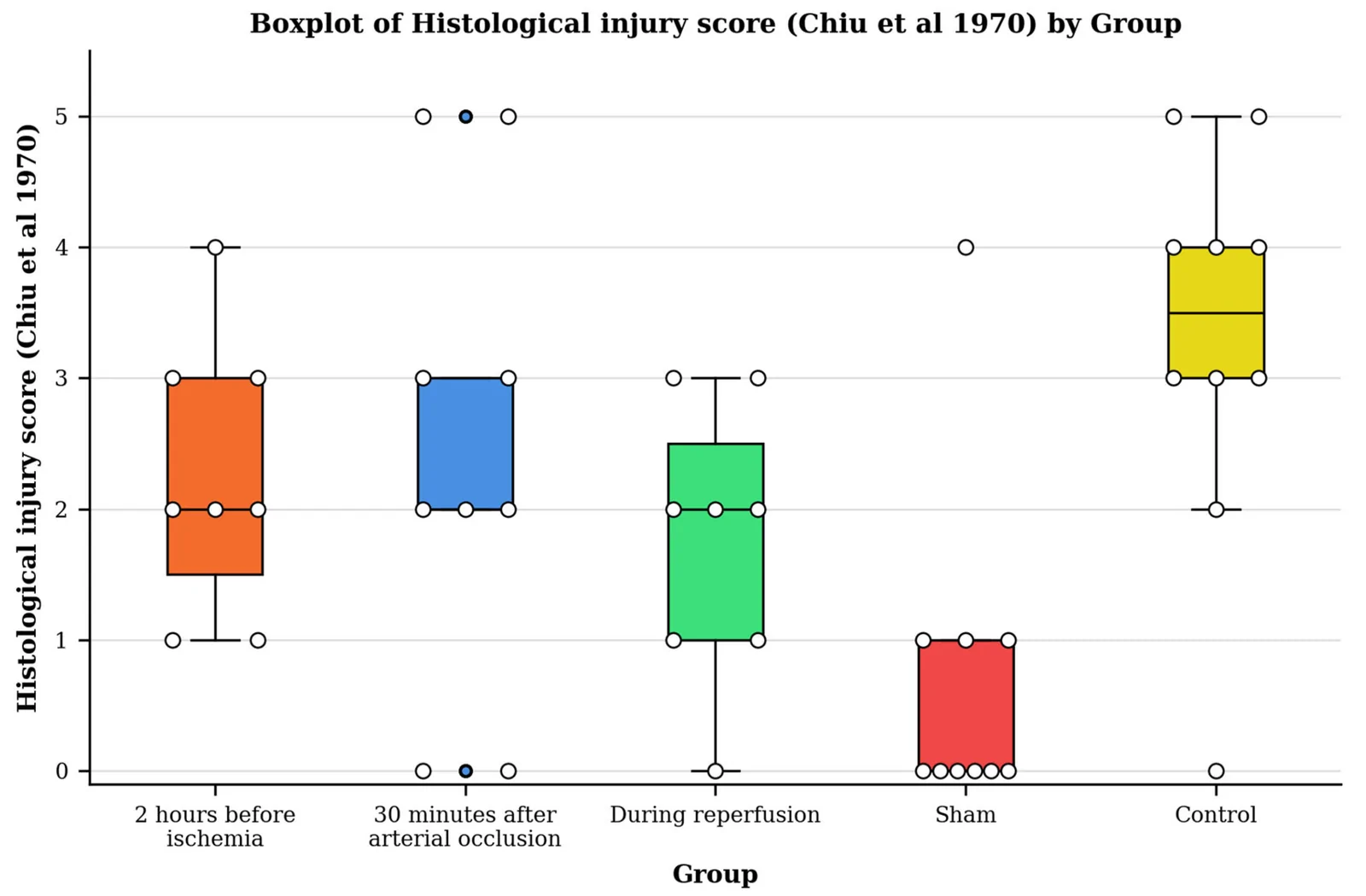
Distribution of Chiu histological injury scores (0–5) by experimental group. Individual observations are shown as open circles; colors distinguish the experimental groups [16].

Median (IQR) Chiu scores are presented in Table 2. The Control group exhibited the greatest mucosal injury (median = 4, IQR = 3–4), while the Sham group had minimal or no histological alterations (median = 0, IQR = 0–1). Median injury scores in all three IGF-I-treated groups were lower than those observed in the untreated Control group, with the lowest median score recorded in the reperfusion group (median: 2; IQR: 1–3) (Figure 3).

The Kruskal–Wallis H test showed a statistically significant difference among the five groups, with H(4) = 14.625 and *p* = 0.0055, indicating that at least one treatment condition differed in its effect on intestinal mucosal injury. Pairwise Mann–Whitney U tests with Bonferroni-adjusted *α* = 0.005 were performed to locate group differences (Table 3).

Only the comparison between Sham and Control remained statistically significant after correction (U = 12.5, *p* = 0.003), confirming that ischemia–reperfusion produced marked mucosal injury compared with surgical manipulation alone.

None of the pairwise comparisons between individual IGF-I-treated groups and the untreated Control group remained statistically significant after Bonferroni correction.

### 3.1. Post Hoc Exploratory Analysis of Pooled IGF-I Groups

As a post hoc exploratory analysis, the three IGF-I-treated groups were pooled into a single treatment group to evaluate the overall histological findings associated with exogenous IGF-I administration irrespective of administration timing. This exploratory analysis was performed because the three treatment groups demonstrated similar distributions of histological injury scores in the primary analysis.

The distribution of histological Chiu scores for the pooled IGF-I-treated group did not significantly deviate from normality (Shapiro–Wilk *p* = 0.063), while the Sham group remained non-normally distributed (*p* < 0.001). The Control group did not violate normality (*p* = 0.176). Given the ordinal nature of the data and the non-normal distribution in at least one group, non-parametric analyses were again used.

The Kruskal–Wallis H test indicated a statistically significant difference in mucosal injury across the three groups (H(2) = 13.935, *p* < 0.001), suggesting that at least one treatment condition produced a distinct histological outcome (Table 4).

Post hoc pairwise Mann–Whitney U tests were conducted to identify specific differences between the pooled IGF-I-treated, Control, and Sham groups. After applying the Bonferroni correction for multiple comparisons (adjusted significance threshold *α* = 0.0167), a statistically significant difference was observed between the IGF-I-treated and Sham groups (U = 46.5, *p* = 0.003), as well as between the Sham and Control groups (U = 12.5, *p* = 0.003). Although the comparison between pooled IGF-I-treated animals and the Control group did not remain statistically significant after Bonferroni adjustment (U = 64.5, *p* = 0.025), the pooled IGF-I group demonstrated numerically lower median histological injury scores than untreated controls (Table 5).

**Figure 4:**
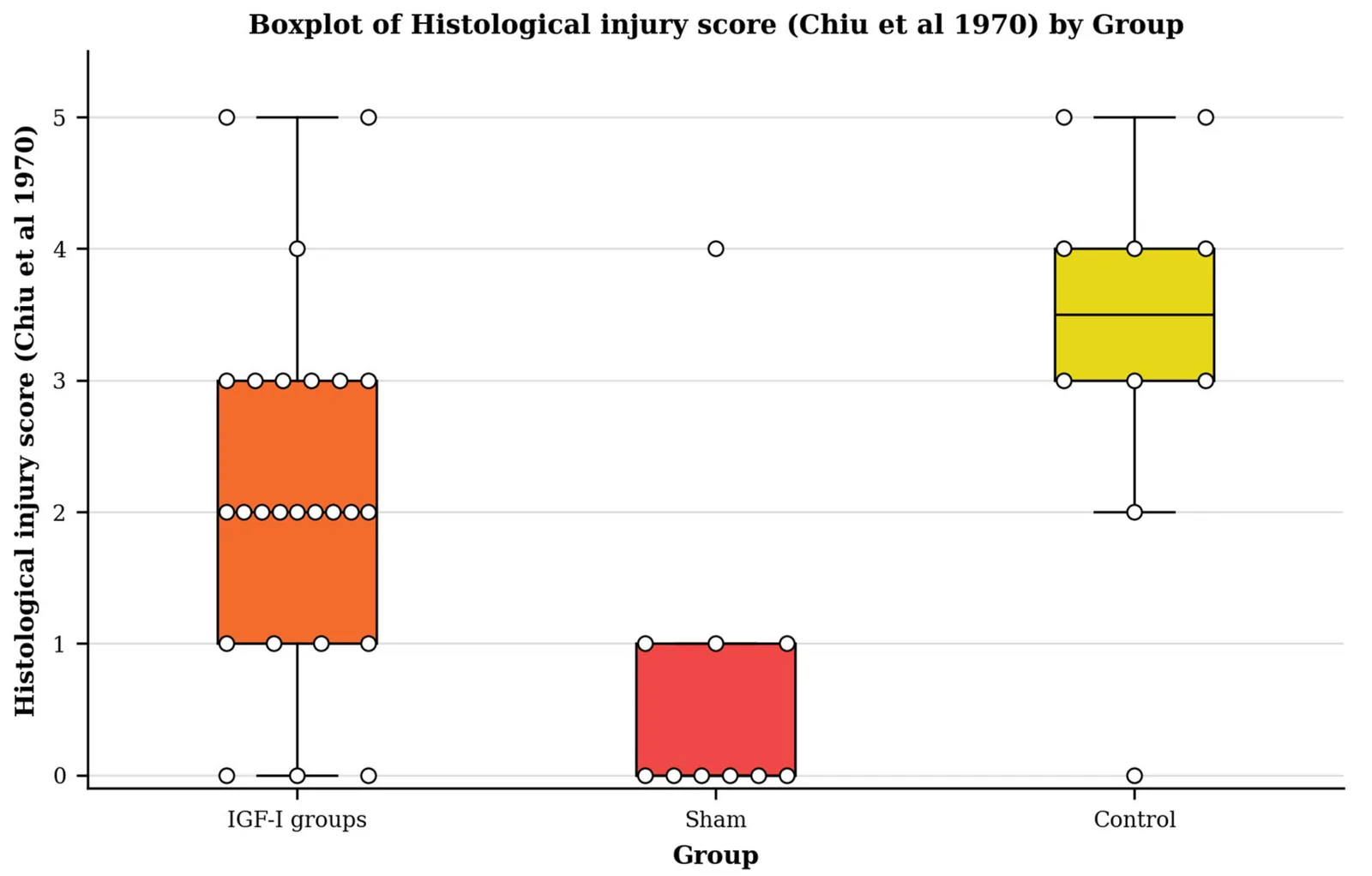
Distribution of Chiu histological injury scores (0–5) by experimental group (pooling all IGF-I groups). Individual observations are shown as open circles; colors distinguish the experimental groups [16].

Median injury scores were lowest in the Sham group (0, 0–1), highest in the Control group (4, 3–4), and intermediate in the IGF-I-treated group (2, 1–3) (Figure 4).

### 3.2. Comparing Only the Three IGF-I Groups

**Figure 5:**
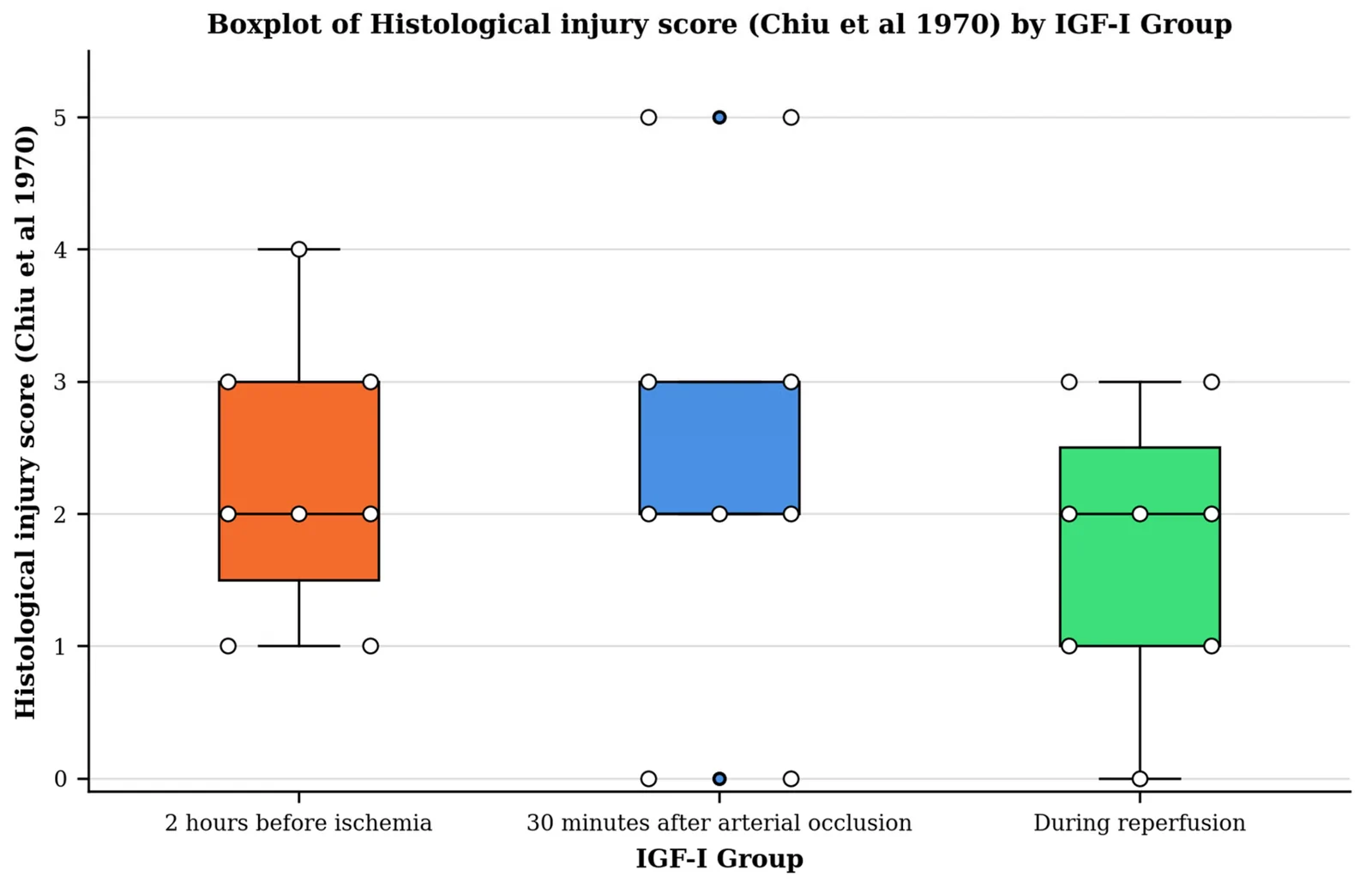
Distribution of Chiu histological injury scores (0–5) by IGF-I group. Individual observations are shown as open circles; colors distinguish the experimental groups [16].

An exploratory comparison was performed among the three IGF-I administration time points to evaluate whether the timing of treatment influenced the degree of intestinal mucosal protection. The Kruskal–Wallis H test showed no statistically significant difference in histological injury among the three groups, with H(2) = 0.959 and *p* = 0.619. Median Chiu scores were identical for the 2 h before ischemia group (2 (2, 3)) and the 30 min after arterial occlusion group (2 (2, 3)), and were similar for the during reperfusion group (2 (1, 3)) (Table 6 and Figure 5).

Overall, the pattern of histological injury was similar across all analyses. The Sham group consistently demonstrated the lowest histological injury scores and the untreated Control group the highest scores, whereas IGF-I-treated animals exhibited intermediate histological injury scores irrespective of the analytical approach.

## 4. Discussion

The present study evaluated the histological effects of IGF-I administration on intestinal mucosal injury following ischemia–reperfusion in a rat model. The ischemia–reperfusion protocol reliably produced marked mucosal damage in untreated controls, while Sham-operated animals showed minimal histological lesions, supporting the validity of the experimental model. Across all analyses, IGF-I-treated animals consistently demonstrated lower median Chiu injury scores compared with untreated controls. However, none of the individual comparisons remained statistically significant after Bonferroni correction. Accordingly, these findings indicate a consistent histological pattern rather than a statistically confirmed treatment effect.

The pathophysiology of intestinal ischemia–reperfusion injury is multifactorial and involves a complex interplay of hypoxia, endothelial dysfunction, inflammatory activation, and regulated cell death. Hypoxia reduces adenylate cyclase activity and intracellular cAMP levels in endothelial cells, leading to increased vascular permeability. Simultaneously, activation of complement pathways and recruitment of inflammatory cells amplify tissue injury, while apoptosis, necrosis, and other regulated cell death pathways contribute to progressive mucosal destruction [17,18]. These complex mechanisms provide the biological rationale for investigating therapeutic interventions aimed at preserving intestinal mucosal integrity during ischemia–reperfusion.

The small intestine exhibits a degree of physiological tolerance to ischemia through autoregulatory mechanisms that preserve oxygen consumption despite moderate reductions in blood flow. Experimental studies have shown that blood flow must decrease by approximately 30–50% before tissue oxygen utilization becomes compromised. Histological injury may become evident after approximately 20 min of complete ischemia, whereas more pronounced disruption of the villous architecture develops with longer ischemic periods, particularly beyond 45 min. Furthermore, enterocyte susceptibility varies according to cellular differentiation and anatomical location along the villus, with mature epithelial cells at the villous tips being particularly vulnerable to ischemic injury [19,20].

The intestinal mucosa is particularly susceptible to ischemic injury because disruption of epithelial integrity and increased microvascular permeability compromise the normal intestinal barrier. Loss of barrier function facilitates the translocation of bacteria and endotoxins into the systemic circulation, potentially resulting in sepsis and multiple organ dysfunction syndrome. Furthermore, both endothelial and epithelial permeability increase progressively with the duration of reperfusion, emphasizing the importance of timely therapeutic interventions aimed at preserving mucosal integrity [21].

The experimental protocol used in the present study was selected to maximize the ability to detect reperfusion-induced mucosal injury while avoiding extensive irreversible ischemic damage. Previous experimental studies have shown that ischemic periods shorter than 20 min produce minimal histological injury, whereas ischemia exceeding 60 min may result in severe irreversible tissue damage that limits assessment of reperfusion-associated injury [22]. Consequently, a 45-min ischemic period was selected. Histological evaluation was performed 3 h after reperfusion, in accordance with the original Chiu model [16], which demonstrated that mucosal injury becomes evident within the first hour of reperfusion and reaches maximal severity after approximately 3 h, a finding that has subsequently been confirmed by other investigators [23].

Several experimental studies have proposed that IGF-I may influence the response to intestinal ischemia–reperfusion injury through multiple complementary biological mechanisms. IGF-I has been shown to promote enterocyte survival and proliferation via activation of the PI3K/Akt signaling pathway, thereby enhancing mucosal regeneration and preserving epithelial integrity [24,25]. In addition, IGF-I exerts anti-apoptotic effects and reduces oxidative stress by modulating the production of reactive oxygen species and upregulating antioxidant defenses [26,27]. IGF-I has also been reported to stabilize the intestinal barrier by maintaining tight junction integrity and limiting bacterial translocation [28,29]. Furthermore, IGF-I may modulate the inflammatory response by downregulating pro-inflammatory cytokines and attenuating neutrophil infiltration [30,31], while improving microcirculatory perfusion through endothelial protection [5,32]. Although these mechanisms are biologically plausible, they were not directly evaluated in the present study, which was designed to assess histological mucosal injury rather than molecular or functional endpoints. Consequently, any mechanistic interpretation of the observed histological findings should be considered speculative.

Despite the well-established biological functions of IGF-I, evidence regarding the therapeutic administration of exogenous recombinant IGF-I in experimental intestinal ischemia–reperfusion injury remains limited [12]. Most published studies have focused on endogenous IGF-I expression, receptor-mediated signaling pathways, or regenerative strategies involving the IGF-I axis, including stem cell-based therapies, rather than direct administration of recombinant IGF-I [13,14,33]. Consequently, direct comparisons among available studies remain challenging because of differences in experimental models, treatment protocols, outcome measures, and timing of administration. Against this background, the present study was designed to provide additional experimental evidence regarding the histological effects of exogenous recombinant IGF-I administered at different phases of intestinal ischemia–reperfusion injury using a standardized experimental model.

In the present study, IGF-I was administered intraperitoneally as a single dose of 200 μg per 100 g of body weight. This route of administration has previously been employed in experimental models of intestinal ischemia–reperfusion, including the study by Haglind et al., while the selected dose was based on comparable experimental protocols [12,34]. The selected dose was intended to provide an adequate single IGF-I exposure while allowing the timing of administration to be evaluated across distinct phases of the ischemia–reperfusion sequence. This study was therefore designed to investigate administration timing using a fixed dose rather than to assess a dose–response relationship. No statistically significant differences were observed among the three administration schedules (pre-ischemia, during ischemia, and at reperfusion). Nevertheless, the similar distribution of histological injury scores across the three treatment groups suggests that, within the conditions of the present experiment, no administration window appears clearly superior. Alternatively, the relatively small subgroup sizes may have limited the ability to detect more subtle timing-dependent differences.

Although experimental evidence regarding intestinal ischemia–reperfusion remains limited, studies in other ischemia–reperfusion models, including cardiac and neural tissues, have similarly suggested that IGF-I may reduce apoptosis, preserve tissue integrity, and facilitate recovery following ischemic injury [35]. Although direct extrapolation across organs should be made with caution, these observations support the biological plausibility of the consistent histological pattern observed in the present study.

As a post hoc exploratory analysis, the three IGF-I treatment groups were pooled to evaluate the overall histological findings associated with exogenous recombinant IGF-I administration irrespective of treatment timing. This analysis demonstrated an overall difference among pooled IGF-I-treated animals, untreated controls, and Sham-operated animals. However, the comparison between pooled IGF-I-treated animals and untreated controls did not remain statistically significant after Bonferroni correction. Furthermore, because the three treatment schedules correspond to biologically distinct phases of ischemia–reperfusion, they should not be considered mechanistically equivalent. Consequently, the pooled findings should be interpreted solely as exploratory and hypothesis-generating.

The present findings should also be interpreted in the context of the inherent limitations of semi-quantitative histological scoring systems such as the Chiu classification. Although widely accepted and validated in experimental intestinal ischemia–reperfusion research, semi-quantitative scoring inevitably involves a degree of observer-dependent interpretation and may be less sensitive than quantitative morphometric or molecular analyses. Furthermore, histological assessment represented the primary study endpoint, while complementary molecular and functional markers were not evaluated, and the relatively small subgroup sizes may have limited the ability to detect subtle timing-dependent differences. Beyond Chiu grading, routine H&E-stained sections may provide additional morphological information, including quantitative assessment of villous architecture and other mucosal morphological features [36,37]. Incorporating such complementary morphological assessments together with molecular and functional biomarkers in future studies may provide a more comprehensive characterization of intestinal mucosal injury and further clarify the biological effects of exogenous recombinant IGF-I. Nevertheless, the use of a standardized experimental model, blinded histological assessment, and consistent findings across complementary statistical analyses provides a solid foundation for future hypothesis-driven investigations.

## 5. Conclusions

In this experimental rat model, animals treated with exogenous recombinant IGF-I consistently demonstrated lower median histological injury scores than untreated ischemia–reperfusion controls. However, these differences did not remain statistically significant after correction for multiple comparisons, and no administration schedule proved superior under the conditions of the present experiment.

Taken together, the present findings provide additional experimental evidence regarding the histological effects of exogenous recombinant IGF-I in intestinal ischemia–reperfusion injury. Although a definitive protective effect could not be established, the consistent histological pattern observed across complementary analyses supported further investigation in larger experimental studies incorporating quantitative histological, molecular, and functional endpoints.

## Figures and Tables

**Table 1:** Chiu grading of the mucosal damage of the intestine after IRI.

Grading Scale	Description
Grade 0	Normal mucosal villi.
Grade 1	Development of subepithelial Gruenhagen’s space, usually at the apex of the villus, and often with capillary congestion.
Grade 2	Extension of the subepithelial space with moderate lifting of the epithelial layer from the lamina propria.
Grade 3	Massive epithelial lifting down the sides of the villi. A few tips may be denuded.
Grade 4	Denuded villi with the lamina propria and dilated capillaries exposed. Increased cellularity of lamina propria may be noted.
Grade 5	Digestion and disintegration of the lamina propria, with hemorrhage and ulceration.

**Table 2:** Median (IQR) Chiu histological injury scores by experimental group.

Group	Chiu et al., 1970 [16], Grading, Median (IQR)	Statistic Kruskal–Wallis
IGF-I, 2 h before ischemia	2 (2, 3)	H(4) = 14.625, *p* = 0.0055 **
IGF-I, during ischemia	2 (2, 3)	
IGF-I, during reperfusion	2 (1, 3)	
Sham	0 (0, 1)	
Control	4 (3, 4)	

** *p* < 0.01.

**Table 3:** Pairwise Mann–Whitney U comparisons with Bonferroni adjustment (*α* = 0.005).

Comparison	Mann–Whitney Statistic
Group I–Group II	U = 33.5, *p* = 0.815
Group I–Group III	U = 24.5, *p* = 0.442
Group I–Group S	U = 10.5, *p* = 0.006
Group I–Group C	U = 20, *p* = 0.083
Group II–Group III	U = 27.5, *p* = 0.423
Group II–Group S	U = 19, *p* = 0.035
Group II–Group C	U = 30.5, *p* = 0.243
Group III–Group S	U = 17, *p* = 0.043
Group III–Group C	U = 14, *p* = 0.021
Group S–Group C	**U = 12.5, *p* = 0.003**

**Table 4:** Median (IQR) Chiu injury scores and Kruskal–Wallis test results for pooled groups.

Group	Chiu et al., 1970 [16], Grading, Median (IQR)	Statistic Kruskal–Wallis
IGF-I group	2 (1, 3)	H(2) = 13.935, *p* < 0.001 ***
Sham	0 (0, 1)	
Control	4 (3, 4)	

*** *p* < 0.001.

**Table 5:** Pairwise Mann–Whitney U comparisons for pooled groups (Bonferroni-adjusted α = 0.0167).

Comparison	Mann–Whitney Statistic
IGF-I group–Sham	**U = 46.5, *p* = 0.003**
IGF-I group–Control	U = 64.5, *p* = 0.025
Sham–Control	**U = 12.5, *p* = 0.003**

**Table 6:** Median (IQR) Chiu injury scores and Kruskal–Wallis test results for IGF-I groups.

Group	Grading: Chiu et al., 1970 [16], Median (IQR)	Statistic Kruskal–Wallis
2 h before ischemia	2 (2, 3)	H(2) = 0.959, *p* = 0.619
30 min after arterial occlusion	2 (2, 3)	
During reperfusion	2 (1, 3)	

## Data Availability

The data presented in this study are available from the corresponding author upon reasonable request.
